# Gut Microbiota and Bipolar Disorder: An Overview on a Novel Biomarker for Diagnosis and Treatment

**DOI:** 10.3390/ijms22073723

**Published:** 2021-04-02

**Authors:** Lorenza Lucidi, Mauro Pettorruso, Federica Vellante, Francesco Di Carlo, Franca Ceci, Maria Chiara Santovito, Ilenia Di Muzio, Michele Fornaro, Antonio Ventriglio, Carmine Tomasetti, Alessandro Valchera, Alessandro Gentile, Yong-Ku Kim, Giovanni Martinotti, Silvia Fraticelli, Massimo Di Giannantonio, Domenico De Berardis

**Affiliations:** 1 Department of Neurosciences and Imaging, Chair of Psychiatry, University “G. D’Annunzio”, 66100 Chieti, Italy; lorenza.lucidi@gmail.com (L.L.); mauro.pettorruso@hotmail.it (M.P.); federica.vellante@gmail.com (F.V.); francesco.dic@hotmail.it (F.D.C.); franca.ceci@live.it (F.C.); Mariachiarasantovito@ymail.com (M.C.S.); dimuzioilenia@gmail.com (I.D.M.); giovanni.martinotti@gmail.com (G.M.); silvieffesc@gmail.com (S.F.); digiannantonio@unich.it (M.D.G.); 2 Department of Psychiatry, Federico II University, 80131 Naples, Italy; dott.fornaro@gmail.com; 3 Department of Psychiatry, University of Foggia, 71121 Foggia, Italy; a.ventriglio@libero.it; 4 NHS, Department of Mental Health, Psychiatric Service for Diagnosis and Treatment, Hospital “Maria SS. dello Splendore” ASL 4, 64021 Giulianova, Italy; carmine.tomasetti@aslteramo.it; 5 Villa “S. Giuseppe” Clinic, Hermanas Hospitalarias, 63100 Ascoli Piceno, Italy; alessandro.valchera@gmail.com; 6 Department of Mental Health, ASREM, 86039 Termoli, Italy; alessandro.gentile@gmail.com; 7 College of Medicine, Department of Psychiatry, Korea University, Seoul 02841, Korea; yongku@korea.edu; 8 NHS, Department of Mental Health, Psychiatric Service for Diagnosis and Treatment, Hospital “G. Mazzini”, ASL 4, 64100 Teramo, Italy

**Keywords:** gut microbiota, bipolar disorder, mood, anxiety, mania, depression

## Abstract

The gut microbiota is the set of microorganisms that colonize the gastrointestinal tract of living creatures, establishing a bidirectional symbiotic relationship that is essential for maintaining homeostasis, for their growth and digestive processes. Growing evidence supports its involvement in the intercommunication system between the gut and the brain, so that it is called the gut–brain–microbiota axis. It is involved in the regulation of the functions of the Central Nervous System (CNS), behavior, mood and anxiety and, therefore, its implication in the pathogenesis of neuropsychiatric disorders. In this paper, we focused on the possible correlations between the gut microbiota and Bipolar Disorder (BD), in order to determine its role in the pathogenesis and in the clinical management of BD. Current literature supports a possible relationship between the compositional alterations of the intestinal microbiota and BD. Moreover, due to its impact on psychopharmacological treatment absorption, by acting on the composition of the microbiota beneficial effects can be obtained on BD symptoms. Finally, we discussed the potential of correcting gut microbiota alteration as a novel augmentation strategy in BD. Future studies are necessary to better clarify the relevance of gut microbiota alterations as state and disease biomarkers of BD.

## 1. Introduction

### 1.1. Bipolar Disorder

Bipolar Disorder (BD) is a severe and chronic mood disorder defined by alternating episodes of mania, hypomania, and depression. It is widely diffused with a prevalence of more than 1% [1].

Manic or hypomanic episodes are characterized by elevated mood and increased activity; they differ in severity and length, with manic episodes being more severe than hypomanic ones. At the onset of the disorder, most patients with BD present a depressive episode, subtly differing from unipolar depressive ones [2].

As to pathogenesis, BD is a highly heritable disorder and a multifactorial model of gene and environment interaction has been proposed [3]. An imbalance in monoaminergic systems, as the serotonergic, dopaminergic, and noradrenergic neurotransmitter systems, plays a key role in the disorder [4]. Neural plasticity seems also to be important in the circuitry regulating affective functions, with neurotrophic molecules, such as brain-derived neurotrophic factor (BDNF), having a vital role in dendritic sprouting and synaptic plasticity [5].

Apart from this evidence, several studies conducted over the last few years have evaluated the possible implications of the gut microbiota in the pathogenesis of BD [6,7].

### 1.2. Composition and Development of the Gut Microbiota

The microbiota represents the set of microorganisms present in an environment [8,9], with the intestinal one including at least 1000 different species. These microorganisms express more than 3 million genes that take the name of the microbiome and which, according to some researchers, represent our second genome [10,11,12].

Bacteria (especially the obligate anaerobes), as well as viruses, protozoa, archaea and fungi, participate in the constitution of the microbiota [10,13]. The bacteria mainly belong to two phyla, Bacteroidetes and Firmicutes [14] and, even if to a lesser extent, the Proteobacteria, Actinobacteria, Fusobacteria and Verrucomicrobia phyla are also present [15].

**Figure 1:**
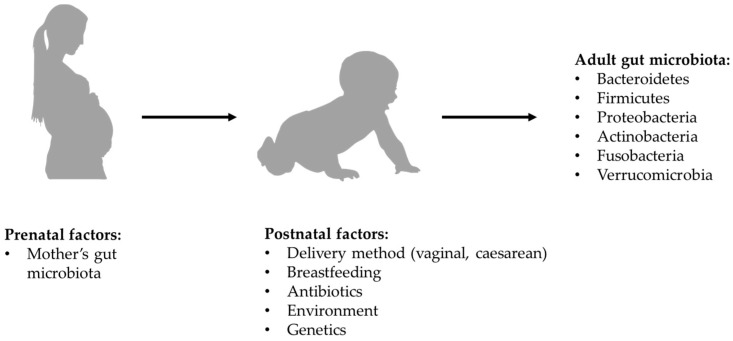
Formation of bacterial component of gut microbiota.

According to most of the current evidence, microbial colonization of the gastrointestinal tract begins at birth, so the way in which delivery is carried out might affect the composition of the microbiota. Specifically, in the case of vaginal birth, the new-born is exposed to the mother’s vaginal microbiota, while in the case of a caesarean section it is exposed to the mother’s skin microbiota [16,17]. However, there is more recent evidence that new-borns begin to acquire their microbiota in utero through the microbes which, crossing the maternal digestive tract, are thought to colonize the digestive tract of the embryo. Regardless of what the exact timing of colonization may be, the composition of the newborn’s microbiota is influenced by maternal diet and lifestyle [18], as well as by the duration of postpartum hospitalization, by the possible use of antibiotics and by the type of breastfeeding. In full-term babies born from vaginal birth and exclusively breastfed, the microbiota has a composition in which beneficial species prevail and *Clostrioides difficile* and *Escherichia coli* are marginally present. After the first 2 weeks of life, the intestinal microbiota seems to acquire relative stability all through the weaning period, with further changes which, although less significant, continue to occur until the 5th year of age [19,20] (see Figure 1).

At this point, the composition of the child’s microbiota is essentially comparable to that of an adult, in which its configuration’s main determinant seems to be the diet [21]. The role of dietary-nutritional factors continues to be influential even in the elderly where, among other things, the composition of the microbiota is strongly correlated to the individual’s health state and levels of frailty [22]. It is believed that the quality of human relationships can also modulate the composition of the microbiota. In this regard, a recent study found that married couples who live in a very close relationship have a very similar composition of the microbiota [23].

### 1.3. Function of Gut Microbiota

The microbiota establishes a symbiotic relationship with the host organism, essential for maintaining homeostasis, as well as growth and digestive processes [24,25]. In detail, the microbiota plays a crucial role in preserving the barrier of the gastrointestinal epithelium, regulating its permeability (through the different expression of proinflammatory cytokines and NF-kB), the luminal absorption of water, the electrolytes and nutrients, and exerting a mechanical defense action against the entry of toxins and pathogens. The bacteria that constitute it modulate the integrity of the narrow intestinal junctions, inhibit the adherence of pathogens to the intestinal barrier, and increase the production of mucin from the goblet cells, the secretory IgA and the secretion of β-defensin in the mucus luminal [26].

The microbiota also appears to be involved in the regulation of various functions of the gastrointestinal lymphoid tissues (GALT) [24]. In support of this last consideration, a study conducted on a large sample of 1,937,488 subjects, 44,259 of which underwent an appendectomy in paediatric age, showed that the latter, compared to controls who have had previous appendicitis, or in healthy conditions or undergoing surgery for hernia abdominal, manifested an increased risk for developing psychological symptoms. Hence, the caecal appendix can act as a reservoir that guarantees the balance of the microbiota in case of dysbiosis [27].

### 1.4. Dysfunctions of Gut Microbiota

The alteration of the symbiotic relationship existing between the microbiota and the host organism takes the name of intestinal dysbiosis and, on the basis of the evidence currently available, it is presumably involved in the development of various gastrointestinal and extra-gastrointestinal disorders; these may include neuropsychiatric disorders. [28,29].

In the specific case of psychiatric disorders, the pathogenetic role of the microbiota could be traced back to its ability to influence the development and the function of the CNS, as well as other relevant behavioral aspects. This ability can be traced back to the modulatory action that the microbiota exerts at the level of the bidirectional communication system that exists between the intestine and the brain, which is called the gut–brain–microbiota axis [30,31].

The mechanisms through which the microbiota performs its function at the level of the aforementioned axis include those of immunological, endocrine, neuronal and vagal autonomic stimulation nature [32].

### 1.5. Aims

Considering the possible implication of microbiota in the development of neuropsychiatric disorders, we focused our attention, reviewing in a narrative way the relevant literature, on the possible correlations between the gut microbiota and BD. Our first aim was to explore pathogenetic links between microbiota and BD and which alterations in the composition of the microbiota itself are associated with the disorder and its different phases. We also reviewed the evidence about the alterations of microbiota linked to the use of psychotropic drugs currently available to treat BD. Finally, the therapeutic potentials of microbiota manipulation for the treatment of BD were reviewed.

## 2. Microbiota and Bipolar Disorder

### 2.1. The Pathogenetic Role of the Gut Microbiota in Bipolar Disorder

Considering the feasible implications of the microbiota in the pathogenesis of BD, it could be regarded as a possible modulator for the inheritance of such disorder. With respect to this, it may be interesting to analyze the study by Vinberg et al. which compared the gut microbiota’s composition in pairs of monozygotic twins, that were discordant and concordant for affective disorders, with that of pairs of monozygotic twins with no history of affective disorders. The results of the aforementioned study showed that sick twins, compared to healthy ones, had less microbial diversity and absence of *Christensenellaceae* family and that these alterations could constitute a vulnerability marker for the development of BD in genetically predisposed subjects [33].

Considering that BD is characterized by the presence of a low-grade inflammatory state [34,35], the microbiota could be involved in the development of such disorder through the modulation of the immune response. In detail, the intestinal microbiota, through the Toll-like receptors (TLRs) is involved in the modulation of the innate component of the immune response. TLRs, expressed on innate response cells and on neurons and glial cells at the CNS level, are involved in the recognition of specific constituents of Gram-positive bacteria and Gram-negative lipopolysaccharide (LPS). The activation of the aforementioned receptors induces the production of pro-inflammatory cytokines (IL-1α, IL-1β, TNF-α, IL-6) which, by exploiting the areas of greater permeability at the level of the Blood–Brain Barrier (BBB), can exercise their action at the level of the CNS [25,36,37]. In relation to this, the presumed role of antibiotic therapy in inducing acute manic episodes in patients with BD should be taken into consideration [38]. Specifically, in a study conducted on 234 patients hospitalized for acute mania, the prescription rate of recent antibiotic therapy was significant and it was associated with greater severity of manic symptoms [39]. The correlation that exists between BD and gastrointestinal inflammatory diseases must also be acknowledged to reinforce the existence of a relationship between microbiota, BD and the immune response. Inflammation is often measured on the basis of the biomarkers of the microbial translocation process. The panel of markers used to diagnose Chron’s disease, for example, includes the detection of antibodies against the *Saccharomyces cerevisiae*, an organism that is part of the normal gut microbiota.

The presence of antibodies against this yeast is probably a response to its presence in the mucous–blood interface, which is compromised in the inflammatory state. More often, BD patients have elevated levels of antibodies against the yeast *Saccharomyces cerevisiae*, especially at a time that is close to the disease onset [40,41]. A further mechanism through which the intestinal microbiota could correlate with the pathogenesis of BD is the alteration of the synaptic pruning process [40]. In humans gut microbiota interferes with microglial cells in the synaptic pruning process during critical periods of development, having a direct effect on neural circuits. Of consequence, some alterations in microbiota composition can change the function of these circuits. Interestingly the aforementioned process occurs simultaneously with the maturation of gut microbiota [42,43]. It has been shown that patients affected by BD present a developmental defect in the synaptic pruning process with consequent anomalies in the modulation of neuronal connectivity at the level of the ventral prefrontal cortex and the limbic cortex [44,45,46,47].

### 2.2. Composition of Gut Microbiota and Bipolar Disorder

While evaluating the composition of the microbiota in patients with BD, the study by Evans et al. (2017), in which the microbiota of patients affected by BD was analysed and compared with that of healthy control (HC) subjects, is of particular interest. Such study showed that the microbiota of the former is characterized by a reduction in *Faecalibacterium*, a Gram-positive microorganism with anti-inflammatory properties, and in an unclassified microorganism belonging to the Ruminococcaceae family. The reduction in *Faecalibacterium* appears to be related to the severity of the disorder, the presence of psychotic symptoms and presumably to alterations in sleep quality [48]. In a similar study, subsequently conducted by Painold et al., in which the reduction of Faecalibacterium in the microbiota of patients with BD was confirmed, a significant increase in organisms classified as belonging to the Actinobacteria phylum and in particular, in the Coriobacteriia class of this phylum. Actinobacteria phylum, such as the Coriobacteriia class are involved in lipid metabolism [49] and therefore are related to cholesterol levels [50]; since BD is frequently associated with metabolic alterations, this could be the reason for their greater representation in patients with BD.

On the other hand, considering the relationship BD-increased risk of obesity [51], it was found that the microbiota of patients with higher BD and Body Mass Index (BMI) harboured a significantly greater quantity of lactobacilli than the group with lower BMI; moreover, the Lactobacillaceae family and the genus Lactobacillus were more abundant in the microbiota of BD patients with metabolic syndrome. Therefore, lactobacilli could be a contributing factor to obesity in BD. The increase in members of the Lactobacillaceae family, together with those of Streptococcaceae and Bacillaceae, was also associated with an increase in IL-6 levels, supporting the correlation between microbiota and inflammatory response. Furthermore, the study found a negative correlation between the alpha diversity of the gut microbiota and the disease duration [49]. However, in the 2019 study by Coello et al., which compared the microbiota of newly diagnosed BD patients with that of first degree relatives without BD and with that of HC subjects, a reduction of Faecalibacterium in the microbiota of patients with BD, highlighted by previous studies, and in those of patients affected by Major Depressive Disorder (MDD) [52], was not found.

Instead, an association has been reported between Flavonifractor, a bacterial genus responsible for the breakdown of quercetin (a flavonoid with antioxidant and anti-inflammatory properties) [53,54] and BD, especially in female smokers. Therefore, the presence of Flavonifractor could influence oxidative stress and inflammation through the breakdown of quercetin. This may reinforce the results of previous studies showing an increase in oxidative stress [55] and the presence of low-grade inflammatory status [56] in patients with BD. Based on the results of the study in question, it was not possible to conclude that Flavonifractor contributes to the increase in oxidative stress and inflammation; hence, in order to clarify a possible role, further investigations on the link between intestinal microbiota and oxidative stress are necessary. The microbiota of the affected patients’ first-degree relatives was comparable to that of healthy subjects [57]. Consistent with the above study, the one by QiaoQiao Lu et al. (2019) did not find any reduction of Faecalibacterium in the microbiota of patients with BD when compared to healthy control (HC) subjects, identifying in the former, among other things, a significant increase in *Faecalibacterium prausnitzii* (producer of butyrate), as well as in *Bacteroides-Group Prevotella, Atopobium Cluster, Enterobacter* spp. and *Clostridium Cluster IV* (manufacturer of butyrate) [11].

In the study conducted by McIntyre et al. in the same year, the Clostridiaceae family was quantitatively more present in patients with BD than in HC subjects. Clostridiaceae is a relatively large family of bacteria, containing more than thirty genera ranging from well-known organisms, such as Clostridium, to lesser-known genera, such as Sarcina. The diversity of organisms within this family, however, makes it difficult to identify specific markers that can link the increase in members of the Clostridiaceae family to different disease states. In the same study, Collinsella was more abundant in patients with BD type II (BD-II) than in individuals with BD type I (BD-I) [58].

### 2.3. Composition of Gut Microbiota and Mania

Through our review, it was not possible to identify studies that analyzed possible compositional alterations of the microbiota in bipolar patients during a manic episode. However, there is some evidence showing that bipolar patients in the manic phase have a gastrointestinal barrier with altered permeability, offering the possibility to assume that the aforementioned impairment of integrity could be traced back to compositional alterations of the intestinal microbiota. In this sense, an interesting study is the one conducted by Faruk et al. (2020) [59], on 41 patients with BD (21 in remission and 20 in manic phase) and 41 HC subjects evaluated by the Young Mania Rating Scale (YMRS) and Hamilton Depression Rating Scale (HAM-D) to determine the severity of mania and depression symptoms, respectively. Mean patients’ serum levels of zonulin and claudin-5 were significantly higher than in the HC, with no difference between zonulin and claudin-5 levels between patients with manic episodes and patients in remission. Zonulin and claudin-5 are increased in patients with BD and this finding may highlight the role of intestinal permeability in the pathogenesis of BD.

Similar results were reported in the 2018 study by Rudzki et al. [60], in which patients with BD had higher serum concentrations of IgG directed towards gliadin and deamidated gliadin, when compared to controls. However, there was no difference between patients and the control group in IgA directed towards gliadin and tTG [61]. In a follow-up study, patients with manic symptoms had an IgG rise towards gliadin at baseline, which normalized after 6 months of treatment [62]. In the same study, patients hospitalized during a 6-month follow-up period were more likely to have an IgG increase against gliadin at follow-up.

### 2.4. Composition of Gut Microbiota and Bipolar Depression

Focusing on the depressive phase of the disorder, Hu et al. in 2019 attempted to define the microbiota composition of depressed BD patients before and after quetiapine treatment and to evaluate the association between microbiota and depressive symptoms, also considering the possibility of using the composition of microbiota as a diagnostic marker of disease and prognostic response to treatment. Bacteroidetes were prevalent in untreated depressed BD patients, while Firmicutes were prevalent in HC. In particular, compared to HC, untreated BD patients had a decrease in various butyrate-producing bacteria, including the genera Roseburia, Faecalibacterium and Coprococcus. Butyrate, at the CNS level, can modulate hippocampal function and promote the expression of BDNF, therefore, the reduction of bacteria that produce butyrate in patients with BD could contribute to the pathogenesis of the disease. In regard to treatment with quetiapine, the treated patients were found to be particularly rich in Gammaproteobacteria when compared to those not treated. In evaluating the associations between the composition of the microbiota and depressive symptoms, it was found that the Montgomery–Asberg Depression Rating Scale (MADRS) scores were negatively correlated with the levels of Acetanaerobacterium, Anaerotruncus (belonging to the Ruminococcaceae family) Stenotrophomonas and Raoultella, yet positively with those of Acinetobacter and Cronobacter [63].

Another study by Aizawa et al. [64] examined the association between Bifidobacterium and Lactobacillus counts and affective symptoms in 39 BD patients with bipolar depression (BPD) (13 BD-I; 16 BD-II according to DSM-IV) and 58 HC. Depressive symptoms were assessed using the 17-item version of the HAM-D including the sleep subscale, while the YMRS was used to assess manic symptoms. Of the patients, 33 were receiving drug treatment. Comparisons of Bifidobacterium and Lactobacillus counts between patients and controls revealed no significant differences between the two groups. In the patient group, there was no significant correlation between bacterial count and HAM-D total score or between bacterial count and YMRS total score. The depressive symptoms subscales (i.e., sleep, activity, psychological anxiety and somatic anxiety) were examined separately and a significantly negative correlation was found between Lactobacillus count and sleep (ρ = −0.45, P = 0.01). In this study, no significant differences were found between patients with BD and HC. However, a noteworthy correlation was found between Bifidobacterium and Lactobacillus counts and depressive symptoms, including disturbed sleep. Furthermore, there was a negative correlation between the Lactobacillus count and the severity of insomnia. Therefore, an increase in Lactobacilli may be useful for sleep disorders in BD.

Depressive symptoms of MDD and BPD are very much alike and, for this reason, Rong et al. [65] hypothesized that patients with MDD and BPD could have a similar gut microbiota structure, still significantly different from that of HC. Furthermore, they hypothesized nuanced differences in the gut microbiota between MDD and BPD due to the indistinguishable clinical characteristics of the depressive component. The study was conducted on patients with MMD (# 30), patients BPD (# 31) and HC (# 30) through the analysis of faecal samples. The Gini coefficient was used to assess the inequality in the different microbiota populations: a higher Gini coefficient meant higher inequality. A Gini coefficient value of zero meant absolute equality; conversely, a Gini coefficient of one represented maximum inequality. In this study, the Gini coefficient of the microbiota (Gm coefficient) was calculated from each participant’s cumulative species curve. That is, the Gm coefficient could have been an indicator of the predominance of several dominant bacteria. Gm coefficients were significantly decreased in both MDD and BPD groups. The increase in Firmicutes and Actinobacteria phyla and the decrease in Bacteroides were significant in the MDD and BPD groups. At the genus level, four of the first five enriched genera (Bacteroides, Clostridium, Bifidobacterium, Oscillibacter and Streptococcus) were found to have increased significantly in the MDD and BPD groups compared to HC. The genera Escherichia and Klebsiella showed significant changes only between the BPD and HC groups. At the species level, compared to patients with BPD, patients with MDD had a greater abundance of Prevotellaceae, including *Prevotella denticola F0289, Prevotella intermedia 17, Prevotella ruminicola* and *Prevotella intermedia*. In addition, the abundance of *Fusobacteriaceae, Escherichia blattae DSM 4481* and *Klebsiella oxytoca* was significantly increased, while *Bifidobacterium longum subsp. Infantis ATCC 15,697 = JCM 1222* was significantly reduced in the BPD group compared to the MDD group. As a result, we found that the dominance levels of the predominant bacteria in the MDD and BPD groups were significantly decreased. Furthermore, eight species or subspecies of bacteria showed biomarker potential to distinguish MDD and BPD patients.

MDD is accompanied by higher serum IgM/IgA responses directed towards the LPS of Gram-negative bacteria, suggesting greater bacterial translocation and intestinal dysbiosis; the latter can occur in bipolar BD. There are differences between MDD and BD-I and BD-II in the biomarkers of nitro-oxidative stress associated with intestinal permeability. Comparing the serum IgM/IgA responses directed to the LPS of six Gram-negative bacteria and the IgG responses to oxidized LDL (oxLDL) in 29 BP1 patients, 37 BP2, 44 MDD and 30 healthy individuals it was found that MDD, BP1 and BP2 are accompanied by an immune response due to increased LPS load, while these aberrations in the gut–brain axis are more pronounced in BP1 and melancholia. In fact, the increase in IgM/IgA responses to *Pseudomonas aeruginosa* significantly discriminated patients with affective disorders (MDD and BD) from controls. Patients with BP1 showed higher IgM responses to *Morganella morganii* than patients with MDD and BP2. Patients with melancholy showed higher IgA responses to *Citrobacter koseri* than controls and non-melancholic depression. The total score on the HAM-D was significantly associated with IgA responses to *Citrobacter koseri*. IgG to oxLDL were significantly associated with increased bacterial translocation. Therefore, drugs that protect the integrity of the intestinal barrier can offer new therapeutic opportunities for BP1 and MDD. A study by Zheng et al. [66] performed gene sequencing on stool samples using faecal 16S ribosomal RNA and found a different microbial population in the gut microbiota of healthy subjects when compared to subjects with MDD and to subjects with BPD. In comparison to healthy subjects, individuals with MDD showed altered covarying operational taxonomic units (OTUs) belonging to the Bacteroidaceae family, while for the BPD group they belonged to the Lachnospiraceae, Prevotellaceae and Ruminococcaceae families. Additionally, 26 OTUs, that can distinguish patients with MDD from those with BPD and HC, have been identified. Finally, 4 of those 26 microbial markers are correlated with disease severity in MDD and BPD [67].

Relevant for the analysis of the microbiota in BD patients is the 2019 Bengesser’s [68] study, in which an interesting comparison was made between depressed and euthymic BD patients: the study has found a reduced diversity and variance in the microbiota of subjects depressed with respect to the euthymic ones.

## 3. Psychotropic Drugs and Alterations of Microbiota Composition

Lithium, mood-stabilizing antiepileptics and atypical antipsychotics (AAPs) are FDA-approved drugs for the treatment of BD [69]; there is often the need to resort to polypharmacy to induce symptom remission [70]. On the basis of the evidence provided by preclinical studies, it was possible to determine some alterations in the composition of the intestinal microbiota following treatment with psychotropic drugs. With regard to lithium, in the preclinical study by Cussotto et al. conducted in vitro and in rats, the drug did not demonstrate antimicrobial properties against *Escherichia coli* and *Lactobacillus rhamnosus*; instead, an increase in the amount of Clostridium, Peptoclostridium, Intestinibacter and of the members of Christensenellaceae family was observed in the intestinal microbiota of mice that received such drug [71]. Considering mood-stabilizing antiepileptics, some preclinical studies have shown how these drugs can alter the composition of the intestinal microbiota. In detail, in a model of murine autism induced by valproic acid (VPA), the administration of such drug to pregnant mothers reduced the diversity of the faecal microbiota of the puppies [72] and, referring to the prior lithium study, similarly determined an increase of Clostridium, Peptoclostridium, Intestinibacter and of the members of Christensenellaceae family in the microbiota of the treated rats [71]. If Lamotrigine is a powerful bacterial ribosomal inhibitor of *Escherichia coli*, it is also endowed with antimicrobial activity towards Gram-positive, such as *Bacillus subtilis* and *Staphylococcus aureus* [73,74].

There are numerous clinical and preclinical studies conducted on different AAPs. Morgan et al., in an in vitro study [75], saw the effect of olanzapine on the growth of two commensal bacterial strains. *E. coli NC101* and *Enterococcus faecalis OGIRF* were evaluated in vitro over a range of supraphysiological concentrations (280 to 560 μg/mL). Olanzapine completely inhibited the growth of Escherichia coli at concentrations above 537 ug/mL, while it did not affect its growth at concentrations below that figure. Olanzapine in rats, on the other hand, reduces the level of Bacteroidetes, leading to an increase in that of Firmicutes, causing hyperphagia, increased visceral fat and peripheral inflammation, especially in females [76,77], with the exhaustion of the microbiota following antibiotic treatment; this has been shown to correct the metabolic dysfunction induced by olanzapine and, at the same time, reverse the effects of the antipsychotic on the intestinal bacterial composition [77]. Other preclinical studies conducted by Bahr et al. showed that female mice treated with Risperidone at a dose of 80 μg/day for 2 months exhibited significant weight gain, due to reduced energy expenditure related to an altered gut microbiota [78]. Finally, Cussotto in a study [71] on aripiprazole found how this can induce marked changes in the composition of the microbiota in rats after a 4-week treatment at 20 mg/kg/day with an increased relative dominance of various taxa including Clostridium, Ruminiclostridium, Intestinibacter and *Eubacterium coprostanoligens*.

Flowers et al. conducted a recent study [79] on humans examining the association between the AAPs and the intestinal microbiota. This was a cross-sectional study in which faecal samples from over 100 bipolar patients were collected and analysed using 16S ribosomal sequencing. Participants were divided into two groups: an AAP-treated group and a drug-free group (at the time of faecal sample collection). The AAPs utilized were: clozapine, olanzapine, risperidone, quetiapine, asenapine, ziprasidone, lurasidone, aripiprazole, paliperidone and iloperidone. The bacterial microbiota of patients treated with AAPs and that of those not treated was significantly different: females treated with AAPs showed reduced species diversity compared to females not treated with AAPs, while males did not show significant diversity. The analysis revealed that the members of *Lachnospiraceae* family and the genera Akkermansia and Sutterella showed differences in abundance between the two groups. Although this study provided a critical view of AAP-mediated changes in the gut microbiota, the report did not include information on diet, which is an important environmental factor driving the composition of the gut microbiota. Additionally, the authors observed drug-specific microbiota differences, but it is not known how these translate into functional differences. Some of the preclinical evidence on the relationship between atypical antipsychotics and alterations of the intestinal microbiota have found correspondence in human studies. In this regard, the use of risperidone and secondary weight gain is associated with an alteration in the intestinal microbiota in adolescent males [78].

Furthermore, in a cohort of patients with BD, the AAPs treatment resulted in a reduction of *Alistipes muciniphila* when compared to BD patients not treated with AAPs [79,80].

## 4. Therapeutic Potential of Microbiota Composition Modification in Bipolar Disorder

### 4.1. Dietary-Nutritional Habits

The composition of the microbiota can be modified by a variation in the dietary-nutritional habits of the individual, especially with respect to the quality and quantity of fats, dietary fibers and carbohydrates consumed [81]. Among other things, the quality of the diet seems to be related to the severity of symptoms in patients with psychiatric disorders [82,83]. Therefore, by modifying the dietary-nutritional habits of the patients, it would be possible to mutate the composition of their intestinal microbiota, presumably improving the symptomatologic picture and their quality of life. Studies conducted in this sense have shown that, in patients with BD, nutritional dietary interventions improve the response to drug therapy, that antioxidants and vitamin B can be used in addendum to antipsychotic drugs in patients with schizophrenia [47] and that diets, particularly rich in terms of fibers contained in fruit and vegetables, correlate with a lower risk of developing depressive symptoms [24,84] by increasing the production of Short-Chain Fatty Acid (SCFA) through colic bacteria. Moreover, SCFAs have positive effects on neurogenesis, synaptic plasticity and cognitive processes [85].

### 4.2. Probiotics

According to the definition of the Food and Agriculture Organization of the United Nations, probiotics are those “live microorganisms which, if administered in adequate quantities, confer a health benefit to the host” [86]. Probiotics are present in some foods, such as yogurt, or are produced in the form of food supplements. They exert their beneficial action on the health of the host by modulating the composition of the microbiota and by helping to maintain the integrity of the intestinal barrier, thus preventing microbial translocation from the luminal compartment to the systemic circulation and therefore the activation of the immune response [87].

The term “psychobiotics” was coined in reference to those probiotics which, presumably modulating bidirectional signaling between the gut and the brain at the level of the gut–brain–microbiota axis, can exert beneficial effects in the additional treatment of patients with psychiatric disorders [15,88,89]. Referring to the results provided by the preclinical and clinical studies, it is important to underline that most of these were conducted using the microorganisms of the genera Lactobacillus and Bifidobacterium as probiotics [47].

As for the preclinical studies, it has been specifically shown that the administration of *Bifidobacterium Infantis*, determining an increase in BDNF at the hippocampal level, is able to reduce the increment in stress response shown by the GF rats, as well as the depressive symptoms exhibited by adult rats exposed to maternal separation during the neonatal period [90]. Regarding the beneficial effects of the genus Lactobacillus, an interesting study is the one by Liang et al. conducted on adult GF rats; these underwent 21 days of restraint stress and were divided into two groups of daily treatment with *Lactobacillus helveticus NS8* and citalopram, following which behavioral tests and biochemical analyses were performed. The results showed that *Lactobacillus helveticus NS8* improved the cognitive and behavioral dysfunctions induced by the stress associated with chronic restraint (anxiety and depression), producing a similar or even greater effect than citalopram, as well as an increase in the expression of hippocampal BDNF mRNA [91]. Furthermore, in a subsequent study, it was shown that a 10-day administration of *Lactobacillus Rhamnosous* is able to normalize anxious behavior in rats [92].

Considering, in regard to BD, the clinical evidence on the potential of additional treatment with probiotics in patients with psychiatric disorders, the results of an uncontrolled pilot study conducted on patients in a euthymic phase showed a slight improvement in cognitive performance after 3 months of supplementation with a probiotic based on nine different Lactobacillus or Bifidobacterium strains [85]. A similar study also found a reduction in ruminant thoughts and in symptoms of the rating scales that measure manic manifestations [85]. Along the same lines, the results of a work conducted on 66 patients recently hospitalized for manic episodes and randomly assigned to a post-discharge probiotic (Lactobacillus/Bifidobacterium) or placebo treatment for 24 weeks, demonstrated a significantly lower re-hospitalization rate for those patients that underwent probiotic supplementation [93].

In support of the possibility of integrating the treatment of mood disorder patients with probiotics, there are several studies that have shown their beneficial effects on anxiety-depressive symptoms. Specifically, a double-blind study conducted on 124 participants, treated for 3 weeks with a mixture of probiotic milk containing Lactobacillus or with placebo, proved that the consumption of probiotics had beneficial effects on mood [94]. Moreover, a study conducted on 55 participants, randomly assigned to 30-day treatment with a probiotic mixture composed of *Lactobacillus helveticus R0052* and *Bifobacterium longum R0175* or with placebo, showed a reduction in Hopkins Symptom Checklist (HSCL-90) scores and anxious-depressive symptoms in patients who received the probiotic mixture [95]. Furthermore, a recent meta-analysis of randomized controlled trials, conducted on patients with MDD, showed that probiotics actually have beneficial effects on symptoms [96].

### 4.3. Prebiotics

Prebiotics, by selectively stimulating the growth and/or activity of a limited number of intestinal microbial species, seem to exert a beneficial effect on the health of the individual [97,98]; galacto-oligosaccharides (GOS), in particular, also appear to act in the brain [99].

Specifically, the results of preclinical studies on mice showed that GOS induced an increase in the expression of BDNF mRNA at the hippocampal level and in the *N*-methyl-d-aspartate receptor subunits in the frontal cortex, presumably inducing an increased proliferation of faecalis Bifidobacterium [100]. Furthermore, when combined with polydextrose, they seem to reduce anxiety levels [24]. In clinical studies conducted on healthy subjects, the administration of GOS appears to be able to counteract the nocturnal awakening from increased cortisol release, as well as the hypervigilance responses induced by the acquisition of negative information [101].

### 4.4. Faecal Transplant

Faecal microbiota transplantation (FMT), i.e., the transfer of faecal material between a donor and a recipient, allows the transfer of the donor’s microbiota to a receiving host and represents one of the currently available options for modifying someone’s microbiota [102,103].

A study by Shunya Kurokawa et al. examined the effect of FMT on psychiatric symptoms, including sleep, among 17 IBS patients with functional diarrhea and functional constipation. The authors measured: changes in the HAM-D and in the sleep-related items’ subscale, in the Hamilton Rating Scale for Anxiety (HAM-A), in the Quick Inventory for Depressive Symptoms (QUIDS), between baseline and four weeks after FMT, and, lastly, the relationship with the intestinal microbiota. At baseline, 12 out of 17 patients were rated with HAM-D ≥ 8. A significant improvement in HAM-D total and sleep subscale score, HAM-A and QUIDS was observed. Moreover, microbiota showed lower diversity in patients with HAM-D ≥ 8 compared to those of healthy donors and to patients with HAM-D < 8. Finally, there was a significant correlation between baseline microbial diversity and the HAM-D score, together with a correlation between microbial diversity increase and improvement in HAM-D after FMT [104].

**Figure 2:**
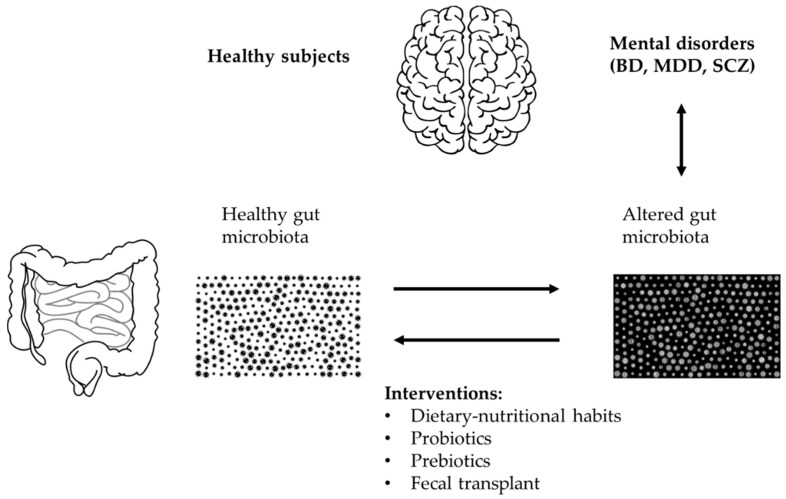
Overview of potential therapeutic intervention for microbiota restoration in bipolar disorder (BD).

A case report investigated the effects of FMT in a 29-year-old patient with BD who, after being diagnosed with type 1 BD in 2012, had undergone numerous hospitalizations and had been treated with numerous drug therapies with little or no benefit; her weight had also increased considerably. In 2016, following the advice of the psychiatrist Russell Hinton, she performed nine FMTs over 11 months and stopped therapy. The donor was her husband who was of normal weight and had a negative psychiatric history. The last depressive episode occurred in March 2017 and the last manic episode in September 2017. In 2019 the patient had lost 33 kg and had no more symptoms [105]. See Figure 2 for an overview of potential therapeutic strategies.

## 5. Discussion

Considering that the most recent evidence underlines the presumable involvement of intestinal dysbiosis in the development of some neuropsychiatric disorders, it appeared of pivotal importance to review the current literature in order to highlight the presence of any correlations between intestinal microbiota and BD. For this reason, we first focused our attention on studies that would highlight the possible pathogenetic implications of the microbiota in the development of the aforementioned disorder, through the modulation of the bidirectional communication system that exists between the intestine and the brain at the level of the microbiota–intestine–brain axis. In the same direction, we also considered the evidence that shows, in BD patients’, some alterations in the composition of the microbiota. A further objective of our review was to evaluate, mainly on the basis of the preclinical evidence currently available, the presence of any correlations between the intestinal microbiota and the drugs used for the treatment of BD, such as lithium, antiepileptic mood stabilizers and atypical antipsychotics. Another objective of our review was to evaluate the currently available evidence on the possibility of improving the response of BD patients’ to psychopharmacological treatment through interventions that induce modification of the intestinal microbiota, such as dietary-nutritional ones and those involving the administration of probiotics and prebiotics.

As regards the first objective of our review, since the microbiota can alter the synaptic pruning process through interaction with microglial cells during critical development periods and, having been shown that BD patients’ can present an anomaly in the aforementioned process during the same periods of development, it is possible that the resulting alterations in cortical and limbic neuronal connectivity may be due to a defective interaction between microglia and intestinal microbiota [47]. Furthermore, being BD characterized by the presence of a low-grade inflammatory state [34,35] it is probable that the microbiota plays its pathogenetic role through the activation of TLRs in the innate component of the immune response [25,36,37]. In support of such pathogenetic hypothesis, it is important to consider, first of all, the study of Coello and collaborators, in which it was detected an association between Flavonifractor, responsible for the degradation of quercetin and BD. In fact, Flavonifractor could presumably influence oxidative stress and inflammation, which we know to be increased in patients suffering from BP [57]. In the same direction, we must consider the correlation between BD and chronic inflammatory gastrointestinal diseases and the presumable role of antibiotics in inducing acute manic episodes in bipolar patients through the alteration of the composition of the intestinal microbiota [39,44,45,46,47].

As to the possible correlation between intestinal dysbiosis and BD, although the studies considered highlighted some differences in the composition of the microbiota of patients with BD compared to HC subjects, they however showed conflicting results. Indeed, although there is evidence supporting a quantitative reduction of butyrate-producing bacteria in the microbiota of BD patients’, later studies have not been able to confirm this finding (although they highlight compositional differences in the microbiota of BD patients compared to HCs) [48,49,57,63,106]. It seemed important to us to focus our attention on the studies that provide evidence of how the microbiota of BD patients is indeed characterized by a quantitative reduction of butyrate-producing microorganisms. This is because, being the latter involved in the modulation of hippocampal function and in the expression of BDNF at the level of the CNS, the decrease in the bacteria responsible for its production could be correlated with the development of the disease. Very striking, in this context, was the study of Hu et al. conducted on depressed bipolar patients, which showed, in their microbiota, a prevalence of Bacteroidetes and a significant reduction of various butyrate-producing bacteria [63]. Furthermore, the 2017 study by Evans et al. was quite innovative: while comparing the composition of the microbiota of DB patients and HC subjects, the researchers have found that the microbiota of the former is characterized by the reduction of Faecalibacterium and that the aforementioned reduction seems to be presumably related to the severity of the clinical picture, the presence of psychotic symptoms, as well as to an impairment in the quality of the sleep–wake rhythm [48]. This same compositional alteration was subsequently confirmed by a similar study that displayed, in regard to the microbiota of bipolar patients, a significant increase in organisms classified in the Actinobacteria phylum and in the class of Choriobacteria. These are microorganisms involved in the processes of lipid metabolism which are, among other things, frequently altered in patients with BD. Furthermore, the same study also demonstrated that bipolar patients with higher BMI had a particularly rich in lactobacilli microbiota, which could presumably contribute to the development of obesity and, consequently, of a metabolic syndrome [49].

It would therefore be interesting to investigate and confirm, through future studies, if indeed the microbiota of BD patients is characterized by a quantitative reduction of the aforementioned microorganisms. In fact, this type of study could allow both to consider this reduction as a possible biomarker of disease and to clarify a further pathogenetic mechanism through which the microbiota could intervene in the development of BD (reduction of butyrate-producing microorganisms-reduced expression of BDNF a CNS level). If the aforementioned compositional alteration were confirmed, it would also be possible to start evaluating the usefulness of integrating the treatment of the aforementioned disorder with interventions capable of restoring the quantity of butyric acid-producing microorganisms.

In reviewing the recent literature that specifically evaluated the composition of the gut microbiota of patients with BPD, we further focused our attention on those that considered patients with MDD, in order to identify if two essentially superimposable disorders, in terms of clinical manifestations, could instead correspond to specific compositional alterations in the intestinal microbiota. In this regard, the study by Rong and collaborators showed that the composition of the microbiota of patients suffering from MDD and BPD is characterized by alterations at the level of phyla Firmicutes, Actinobacteria and Bacteroidetes, with depressed bipolar patients presenting a significant increase of Escherichia and Klebsiella and patients with MDD presenting a greater abundance of the members of Prevotellaceae family [65]. In a similar study, conducted by Zheng et al., it was also found that patients with MD had an altered composition at the level of members of the Bacteroidaceae family, while those affected by BPD presented alterations at the level of members of the Lachnospiraceae, Prevotellaceae and Ruminococcaceae families [67].

As to the second part of our review, is of critical importance to understand if alterations following psychopharmacological therapy for BD can be considered biomarkers of response, non-response and the possible appearance of side effects. Regarding evidence provided by preclinical studies, lithium and valproic acid seem to induce an increase in Clostridium, Peptoclostridium, Intestinibacter and in the members of Christensellenaceae family in the intestinal microbiota of treated mice [71]. As for the atypical antipsychotics, there seems to be a correlation between the administration of olanzapine, risperidone and aripiprazole and alterations in the composition of the microbiota of rats subjected to pharmacological treatment [76,77,78]. In the specific case of olanzapine, through a presumable reduction in the quantity of Bacteroidetes with a consequent increase in that of Firmicutes, it seems to determine, especially in females, hyperphagia, an increase in visceral adipose tissue and peripheral inflammation. The fact that in female rats the dysmetabolic effects of olanzapine can be supported by alterations in the composition of the microbiota seems to be confirmed by the evidence that, by administering an antibiotic treatment causing exhaustion of the same microbiota, the dysmetabolic picture induced by the antipsychotic drug is normalized [77]. Additionally, risperidone treatment, presumably inducing alterations in the composition of the microbiota, appears to be related to weight gain in treated female rats [78]. In clinical studies, risperidone treatment and secondary weight gain were associated with an alteration of the intestinal microbiota in adolescent male subjects [78]. A further study conducted on humans has shown, among other things, that treatment with various atypical antipsychotics such as clozapine, olanzapine, risperidone, quetiapine, asenapine, ziprasidone, lurasidone, aripiprazole, paliperidone and iloperidone, would induce compositional variations in the microbiota of the female subjects undergoing treatment [79].

Further studies are necessary to confirm what role the compositional alterations of the microbiota that occur during pharmacological treatment play in the response to the treatment itself and in the appearance of side effects. Further investigations of this type could also make it possible to identify the presence of specific alterations that could be uniquely associated with a specific pharmacological treatment. These data would bring interesting implications in clinical practice, providing psychiatrists with an additional tool to draw on in case there is the need to evaluate why a patient does not respond to a certain psychotropic agent and/or because he manifests certain side effects. In the same desirable direction, it would therefore be possible to modify psychopharmacological therapy on the basis of understanding highly specific biomarkers.

As regards the last part of our review, interesting results have emerged on those intervention modalities that involve modulation of the microbiota. In particular, through interventions that improve the quality of the diet of psychiatric patients, including bipolar ones, it seems possible to implement the response to drug therapy and to obtain a beneficial effect on depressive and cognitive symptoms [47]. As for the integration with probiotics, several preclinical and clinical studies have shown how the microorganisms of the genera *Lactobacillus* and *Bifidobacterium* can exert beneficial effects on psychiatric symptoms, so much as to prompt the introduction of the term “psychobiotics”. In more detail, considering the results provided by the preclinical studies, it seems that treatment with the aforementioned probiotics is able to reduce the responses to stressful events, as well as to anxiety and depressive symptoms [90,91,92]. By evaluating the evidence provided by clinical studies, specifically addressing patients with BD, it appears that the additional treatment with probiotics can improve cognitive and obsessive symptoms, as well as reduce manic exacerbations of the disorder [93,94,95,96,101,107]. In the same direction, the study by Aizawa et al. highlighted a relationship between the counts of Bifidobacterium and Lactobacillus and depressive symptoms in bipolar patients, with an important correlation between the Lactobacillus count and the severity of insomnia; this offers the possibility of using probiotics based on lactobacilli for treatment of sleep disorders in this category of patients [64] and provide interesting evidence of how treatment with “psychobiotics” can have beneficial and ameliorative effects on various symptomatic aspects of BD.

Even preclinical and clinical evidence on prebiotic supplementation based on GOS have provided interesting results respectively in terms of induced an increase in the expression of BDNF mRNA at the hippocampal level and improvement of anxious and depressive symptoms and sleep disturbances. For these reasons and because the reduction of various butyrate-producing bacteria is probably involved in the reduction of BDNF expression at the hippocampal level [63], it seems useful to suggest future research to further evaluate the possibility of implementing BD patients’ treatment response through prebiotics.

It would be helpful for such evidence to be further investigated and confirmed through future studies so that clinicians can effectively consider probiotics and prebiotics as additional therapeutic tools available for the management of BD. The possibility of having non-invasive treatments of this type available, with potential beneficial effects on the symptoms of patients with BD, would, in our opinion, be particularly useful for the management of a serious, complex disorder subject to frequent exacerbations.

In the context of the intervention that provides for the modification of the intestinal microbiota, it seemed appropriate to mention FMT as well. Specifically, we referred to the case of a patient suffering from BD-I: the subject was poorly responsive to drug therapy and, after performing nine FMTs over an 11-month period and following the suspension of psychopharmacological therapy, she presented a considerable weight decrease and a significant improvement in symptomatology [105]. Obviously, even in this case, we shall not ignore the need for further investigations in order to be able to consider FMT an additional therapeutic option for patients suffering from BD.

## 6. Conclusions

Through our review of the relevant literature, we can assume that there are correlations between BD and the gut microbiota. In this sense, it seems important to underline the need for future investigations that allow us to further understand the pathogenetic implications of the microbiota in the development of the disorder. Even more important there seems to be the need to perform other studies that make it possible to identify which compositional alterations of the microbiota can be considered biomarkers for the diagnosis of BD and to evaluate the response to drug therapy, as well as its correlation with drug-related side effects. Additionally, considering the beneficial effects of the interventions that involve the manipulation of the microbiota on mood, anxiety, sleep, cognition and behavior, future research will contribute to determining the real role of these interventions to improve augmentation strategies, thus expanding current pharmacological, psychoeducational and psychotherapeutic tools for the treatment of BD.

## Data Availability

Not applicable.
